# Dataset on the microstructure Ni_50_Mn_38_Sb_9_Si_3_ alloy and compositions of Ni_50_Mn_38_Sb_12−*x*_Si*_x_* (*x*=2.5, 3) ferromagnetic shape memory alloys

**DOI:** 10.1016/j.dib.2018.05.002

**Published:** 2018-05-09

**Authors:** Ruochen Zhang, Xuexi Zhang, Mingfang Qian, Jianfei Sun, Lin Geng

**Affiliations:** School of Materials Science and Engineering, Harbin Institute of Technology, Harbin 150001, China

## Abstract

The data presented in this article is the supplementary data of Zhang et al. (2018) [1]. The Ni_50_Mn_38_Sb_9_Si_3_ alloy is annealed at 1223 K for 24 h and then quenched into ice water; while the Ni_50_Mn_38_Sb_9.5_Si_2.5_ alloy is annealed at 1173 K for 24 h and then quenched into ice water. The microstructure of the Ni_50_Mn_38_Sb_9_Si_3_ alloy indicates that a higher heat treatment temperature cannot prevent the formation of secondary phases. Furthermore, the composition of α phase is similar to the nominal composition of the alloy. On the other hand, the nominal concentration of Si atoms and heat-treatment temperature do not affect the compositions of the β and γ phases. For example, the compositions of the β and γ phases in the Ni_50_Mn_38_Sb_9_Si_3_ alloy are similar when annealed at 1223 K for 24 h and 1173 K for 24 h

**Specifications Table**

**Table:** 

Subject area	Physics
More specific subject area	Materials Science
Type of data	Tables, Figures
How data was acquired	SUPRA55 scanning electron microscope (SEM) equipped with an energy dispersive X-ray spectroscopy EDS
Data format	Raw, Analyzed
Experimental factors	Polycrystalline Ni_50_Mn_38_Sb_12−*x*_Si*_x_* (*x*=2.5, 3) alloy ingots were prepared by vacuum induction melting under a high purity Ar atmosphere. The samples were re-melted two times in order to improve the compositional homogeneity. The ingot of Ni_50_Mn_38_Sb_9.5_Si_2.5_ alloy was annealed at 1173 K for 24 h, and then quenched into ice water. The ingot of Ni_50_Mn_38_Sb_9_Si_3_ alloy was annealed at 1223 K for 24 h, and then quenched into ice water.
Experimental features	The microstructure of Ni_50_Mn_38_Sb_9_Si_3_ alloys were observed by SEM. The compositions of the α, β and γ phases in the Ni_50_Mn_38_Sb_9.5_Si_2.5_ and Ni_50_Mn_38_Sb_9_Si_3_ alloys were examined by SEM-EDS.
Data source location	Harbin, China
Data accessibility	The data are available with this article.

**Value of the data**

• This data fulfills the microstructure of Ni_50_Mn_38_Sb_9_Si_3_ alloy that was annealed at 1223 K for 24 h and then quenched into ice water.
• This data presents the compositions of α, β and γ phases in Ni_50_Mn_38_Sb_9.5_Si_2.5_ and Ni_50_Mn_38_Sb_9_Si_3_ alloys, which were annealed at 1173 and 1223 K for 24 h, respectively, and then quenched into ice water.
• This data are useful in understanding the influence of the nominal concentration of Si atoms and heat-treatment temperature on the compositions of α, β and γ phases in Si-doped Ni-Mn-Sb-Si alloys.

## 1 Data

**Fig. 1:**
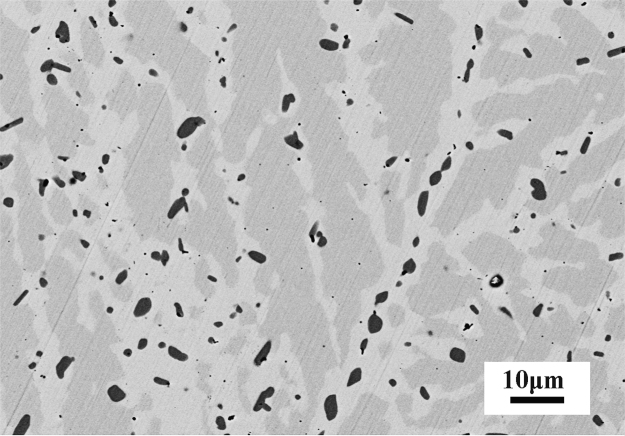
Scanning electron microscope (SEM) image showing the microstructure of Ni_50_Mn_38_Sb_9_Si_3_ alloy annealed at 1223 K for 24 h, and then quenched into ice water.

The dataset of this article provides information on the microstructure of Ni_50_Mn_38_Sb_9_Si_3_ alloy annealed at higher temperature and the compositions of α, β and γ phases in Ni_50_Mn_38_Sb_12−*x*_Si_x_ (*x*=2.5, 3) alloys. Fig. 1 shows the microstructure of the Ni_50_Mn_38_Sb_9_Si_3_ alloy annealed at 1223 K for 24 h and then quenched into ice water. Table 1 shows the compositions of α, β and γ phases in Ni_50_Mn_38_Sb_12−*x*_Si*_x_* (*x*=2.5, 3) alloys at two different heat treatment conditions.

**Table 1:** The compositions of α, β and γ phases in Ni_50_Mn_38_Sb_9.5_Si_2.5_ (NMSS2.5) and Ni_50_Mn_38_Sb_9_Si_3_ (NMSS3) alloys.

Alloys	Phases	Ni (at%)	Mn (at%)	Sb (at%)	Si (at%)
NMSS2.5^a^	α	47.7	42.4	7.7	2.2
	β	45.5	37.9	15.3	1.3
	γ	50.2	35.8	0.8	13.2
Average composition	–	47.0	41.0	9.6	2.4
NMSS3^b^	α	48.7	40.9	7.9	2.5
	β	47.6	38.6	11.7	2.1
	γ	51.1	34.9	0.6	13.4
Average composition	–	48.4	39.5	9.1	3.0

a NMSS2.5 alloy was annealed at 1173 K for 24 h, and then quenched into ice water.

b NMSS3 alloy was annealed at 1223 K for 24 h, and then quenched into ice water.

## 2 Experimental design, materials and methods

### 2.1 The microstructure of Ni_50_Mn_38_Sb_9_Si_3_ alloy and compositions of α, β and γ phases in Ni_50_Mn_38_Sb_12−*x*_Si*_x_* (*x*=2.5, 3) alloys

The heat treatment process of the Ni_50_Mn_38_Sb_12–*x*_Si*_x_* (*x*=2.5, 3) alloys was similar to Ref. [2]. The as-cast ingots were placed in a quartz tube, evacuated and sealed using an oxygen-acetylene flame. Then the ingot of Ni_50_Mn_38_Sb_9.5_Si_2.5_ (NMSS2.5) alloy was annealed at 1173 K for 24 h, and then quenched into ice water. This alloy ingot was used to investigate the effect of Si content on the compositions of α, β and γ phases. The ingot of Ni_50_Mn_38_Sb_9_Si_3_ (NMSS3) alloy was annealed at 1223 K for 24 h and then quenched into ice water, which was used to explore the effect of higher heat treatment temperature on the formation of the α, β and γ phases. The compositions of α, β and γ phases in Ni_50_Mn_38_Sb_12−*x*_Si*_x_* (*x*=2.5, 3) alloys at two different heat treatment conditions were investigated and the influence of the nominal concentration of Si atoms and annealing temperature on the composition of α, β and γ phases in Ni-Mn-Sb-Si alloys was revealed. Fig. 1 showed that higher heat treatment temperature could not prevent the formation of secondary phases (β, γ); however, the quantity of these secondary phases decreased obviously. Table 1 presented that the atomic content of α, β and γ phases in NMSS2.5 and NMSS3 alloy obeyed the same trend as that in NMSS3 alloy, which has been discussed in the article. Additionally, the composition of the γ phase seemed to be independent of the nominal concentration of Si atoms and the heat treatment temperature.

## References

[1] Zhang R.C., Zhang X.X., Qian M.F., Sun J.F., Geng L. (2018). Effect of Si doping on microstructure and martensite transformation in Ni-Mn-Sb ferromagnetic shape memory alloys. Intermetallics.

[2] Zhang R., Qian M., Zhang X., Qin F., Wei L., Xing D., Cui X., Sun J., Geng L., Peng H. (2017). Magnetocaloric effect with low magnetic hysteresis loss in ferromagnetic Ni-Mn-Sb-Si alloys. J. Magn. Magn. Mater..

